# Model-Based Deep Learning PET Image Reconstruction Using Forward-Backward Splitting Expectation Maximisation

**DOI:** 10.1109/nss/mic42101.2019.9059998

**Published:** 2019-10

**Authors:** Abolfazl Mehranian, Andrew J. Reader

## I Introduction

Model-based image reconstruction of positron emission tomography (PET) has now almost superseded conventional reconstruction methods by accounting for all statistical and physical processes of data acquisition in the image reconstruction. Founded on a Bayesian framework, these techniques can even model the prior probability distribution of the unknown activity distribution. Different priors have been proposed, particularly to suppress noise in the reconstructed images without compromising image quality. Based on Markov random fields, the majority of these priors aim to assign a low probability to image solutions that have large local intensity differences on the assumption that those differences are due to noise. The major limitation of these hypothesis-driven priors is that they might also suppress legitimate image details and boundaries. Thus, edge-preserving and anatomically informed priors have been used to reduce noise while preserving PET details [1]. However, their performance highly depends on their functional form and hyperparameters, which are often hand-engineered and selected before reconstruction. Deep learning techniques have recently shown promise in image reconstruction [2], denoising and analysis [3, 4]. In particular, deep convolutional neural networks (CNNs) have an immense potential to learn most representative image features from a multi-modal training space and hence give rise to data-driven priors which can surpass hypothesis-driven ones. Moreover, these techniques have been used to learn the whole reconstruction process by mapping measured data into image space. In contrast, model-based deep learning (MoDL) techniques aim to merge the power of model-based Bayesian algorithms with neural networks through unrolling an iterative optimization algorithm, which provides an elegant theoretical foundation for designing robust data correction and image models. In this work, we propose an optimization algorithm for Bayesian PET reconstruction, which generalizes De Pierro’s MAPEM algorithm [5] for any differentiable priors, and unrolls the resulting algorithm into a recurrent neural net (RNN) in which CNNs are used to learn image features while activity images are reconstructed from emission data. Additionally, the regularization parameter is learned, and anatomical side information can also be incorporated without suppressing PET features.

## II Material and Methods

### A Forward-backward splitting expectation maximisation (FBSEM)

The Bayesian maximum *a posteriori* (MAP) reconstruction of PET data is obtained by the following maximisation:

(1) $$\begin{array}{l} \hat{x}=\underset{\text{x}}{\text{argmax}}\{L(y|x)-\beta R(x)\}\\ L(y|x)=\underset{i}{\sum }y_{i}\text{log}\left({\left[Hx\right]}_{i}+{\bar{b}}_{i}\right)-\left({\left[Hx\right]}_{i}+{\bar{b}}_{i}\right) \end{array}$$

where *L* is the Poisson log-likelihood of measured data, ***y***, given an activity distribution, ***x. H*** is a system matrix and $\bar{b}$ is the expected accidental coincidences. *R* is a penalty function that imposes prior information about ***x***, controlled by the regularization parameter *β*. Eq. (1) can be solved using optimisation transfer techniques as long as a separable, differentiable and convex surrogate can be defined for *R*. Consequently, a monotonically convergent MAP expectation maximisation (EM) algorithm is obtained [5]. In this work, we use a forward-backward splitting technique [6] for solving Eq. (1) for any differentiable convex prior. As a result, the optimisation is performed in the following steps:

(2) $$x_{Reg}^{\left(n\right)}=x^{\left(n-1\right)}-\gamma \beta \nabla R(x^{\left(n-1\right)})$$

(3) $$x^{\left(n\right)}=prox_{\gamma L}(\text{x})≜\underset{\text{x}}{\text{argmax}}\{L(y|x)-\frac{1}{2\gamma }∥x-x_{Reg\;}^{\left(n\right)}{∥}^{2}\}$$

Eq. (2) is the gradient descent minimization of *R* with the step size of *γ*, whereas Eq. (3) is a proximal mapping [6] associated with the log-likelihood *L* with 1/*γ* as a regularization parameter that controls the data fidelity of ***x*** to ***y*** and its proximity to $x_{Reg\;}^{\left(n\right)}$. The quaratic prior in Eq. (3) is separable. Following [7], a seprarable surrogate is then defined for the function *L*, thereby Eq. (3) is maximised in two steps: Eqs. (5-6) of Algorithm 1, accelerated using ordered subsets (OS).

Algorithm 1 **FBSEM for MAP PET image reconsruction**

**Initialize: *x***^(0,1)^ = **1**, number of iterations (*N*_*it*_) and subsets (*N*_*sub*_)

**For** *n* = 1, …, *N*_*it*_

  **For** *m* = 1, …, *N*_*sub*_

(4) $$x_{j,Reg}^{\left(n,m\right)}=x_{j}^{\left(n-1,m\right)}-\gamma \beta \frac{\partial }{\partial x_{j}}R\left(x^{\left(n-1,m\right)}\right)$$

(5) $$x_{j,EM}^{\left(n,m\right)}=\frac{x_{j}^{\left(n-1,m\right)}}{s_{j}^{\left(m\right)}}\underset{i\in {\text{Ω}}_{m}}{\sum }\frac{h_{ij}y_{i}}{\Sigma_{k}h_{ik}x_{j}^{\left(n-1,m\right)}+{\bar{b}}_{i}},\;s_{j}^{\left(m\right)}=\underset{i\in {\text{Ω}}_{m}}{\sum }h_{ij}$$

(6) $$x_{j}^{\left(n,m\right)}=\frac{2x_{j,EM}^{\left(n,m\right)}}{\left(1-\delta_{j}x_{j,Reg}^{\left(n,m\right)}\right)+\sqrt{{\left(1-\delta_{j}x_{j,Reg}^{\left(n,m\right)}\right)}^{2}+4\delta_{j}x_{j,EM}^{\left(n,m\right)}}},\;\delta_{j}=\frac{1}{\gamma s_{j}^{\left(m\right)}}$$

  **End**

**End**

Hence, the optimisation of Eq. (1) is ‘split’ into three steps: *regularization* of the previous image estimate, *EM update* of the previous image estimate and *fusion* of the resulting two images, weighted by *γ* and the subset-dependent sensitivity image ***s***^(*m*)^. Algorithm 1 can be used for the following commonly used Tikhonov prior, weighted by MR information (*w_jb_*):

(7) $$R(x)=\frac{1}{2}\underset{j}{\sum }\underset{b\in N_{j}}{\sum }w_{jb}{\left(x_{j}-x_{b}\right)}^{2}$$

**Fig. 1:**
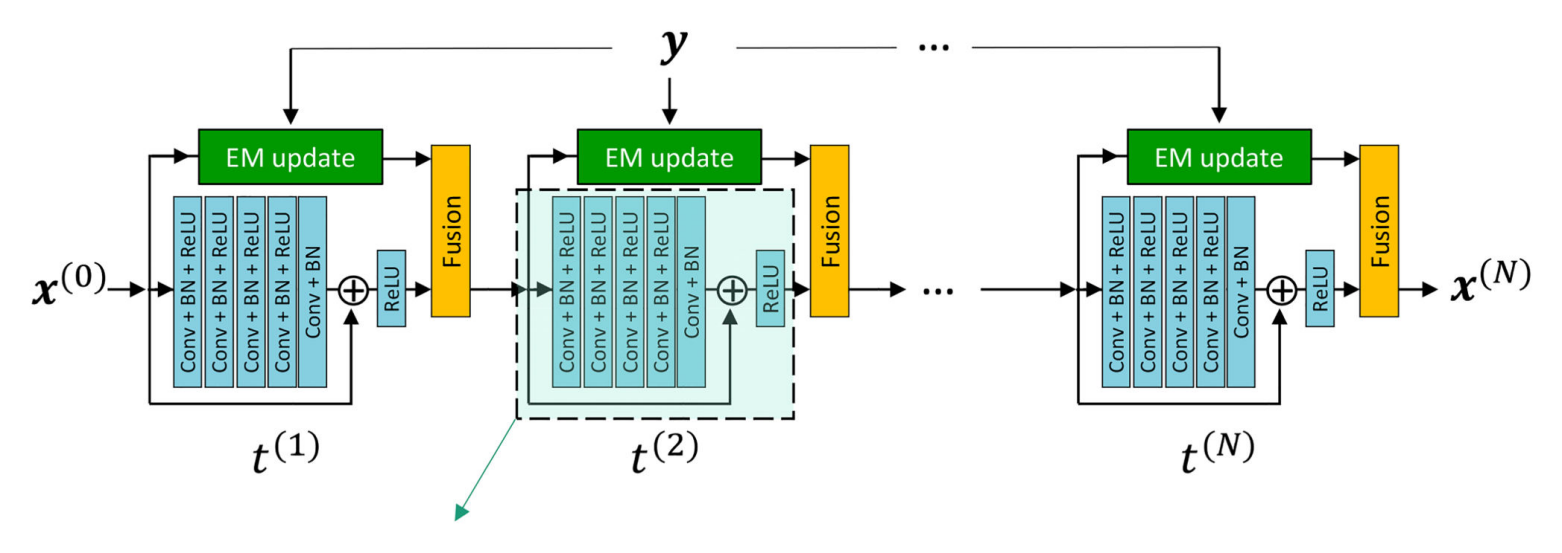
Architecture of the proposed network using a residual learning unit with 5 layers of 32 convolution (Conv) filters, batch normalization (BN) and a regularization parameter, leading to a total of *d* = 28612 trainable parameters, which are shared across all reconstruction states (*t*).

For this prior, by setting $\beta =\frac{1}{2},\gamma =\frac{1}{\Sigma_{b}w_{jb}}$ in Eq. (4), we obtain:

(8) $$x_{j,\;Reg\;}^{\left(n,m\right)}=\frac{1}{2\Sigma_{b}w_{jb}}\underset{b\in N_{j}}{\sum }w_{jb}\left(x_{j}^{\left(n-1,m\right)}+x_{b}^{\left(n-1,m\right)}\right)$$

whereby, Algorithm 1 is reduced to De Pierro’s MAPEM algorithm [5]. As *γ* → ∞, this algorithm degenerates to the OSEM algorithm. In this paper, we used a CNN-based model for *R* and unroll the FBSEM algorithm into an RNN with *N* = *N*_*it*_ × *N*_*sub*_ reconstruction states, in which model parameters are shared across all states. As shown in Fig. 1, a 5-layer learning unit with a non-negativity constraint (imposed by a rectified linear unit, ReLU) was used, whereby Eq. (4) was converted to a residual learning unit [4]. Other deep models such as convolutional encoder-decoders (e.g. U-Net [3]) could also be used. The proposed network was trained in a supervised manner using a training dataset composed of *N*_s_ reference high-dose PET images $\left(x_{s}^{Ref}\right)$, low-dose PET sinograms (***y***_s_, ${\bar{b}}_{s}$) and optionally co-registered MR images $\left(x_{S}^{MR}\right)$. The training was formulated as the minimization of the mean-squared-error loss function between network’s output $\left(x_{s}^{\left(N\right)}\right)$ and $x_{S}^{Ref}$:

(9) $$\begin{array}{l} \hat{\theta }=\underset{\theta }{\text{argmin}}\frac{1}{N_{s}}\sum_{s=1}^{N_{s}}∥x_{s}^{\left(N\right)}-x_{s}^{Ref}{∥}^{2}\\ x_{s}^{\left(N\right)}={\text{FBSEM}}_{\theta }\left(y_{s},{\bar{b}}_{s},x_{s}^{MR}\right),\;N=N_{it}\times N_{sub} \end{array}$$

where model parameters, ***θ*** ∈ ℝ*^d^*, include convolution kernels, biases, batch normalization parameters and *γ*. Eq. (9) was optimized using the Adam optimizer (50 epochs, 10 mini-batches and learning rate of 0.05). Thanks to the parallelism of FBSEM net, the EM-update module was implemented in Python, while regularization and fusion modules (with trainable parameters) were implemented in PyTorch, therefore backpropagation only passes through those modules.

### B Simulations, Training and Evaluation

BrainWeb’s brain phantoms were used to generate 2D FDG PET training sets for the Biograph mMR scanner with a resolution of 2.08×2.08 mm^2^. A total of 100 slices were chosen from five brain phantoms. Ten circular cold and hot lesions with random radii of 2-10 mm and random locations were simulated in the PET phantoms. Similar lesions were simulated in corresponding T1-MR images. Each PET phantom (and its MR and attenuation maps) was rotated by 5 random angles (within ±5°), leading to *N_s_* = 500 2D training samples. For each sample, a 100-M count reference image, a 1M-count low-dose sinogram and its OSEM image were generated. For testing, a separate dataset was generated using different brain phantoms with different counts in the range of 0.05-1 M, random rotations of ±10° and random lesions. For comparisons, a MAPEM with Tikhonov prior, guided by MR-informed Gaussian weights [1], was used and reconstructed by the FBSEM algorithm, with *γ* optimized based on a normalized root mean squared error (NRMSE) criterion. For post-reconstruction denoising of low-dose OSEM images, a U-Net (with 4 resolution levels and a total of 839489 trainable parameters) was trained on the unfiltered OSEM images of the same dataset used for FBSEM net. A 4-mm Gaussian filtering of OSEM images was also included. All OSEM and FBSEM reconstructions were performed with *N*_*it*_ = 10 and *N*_*sub*_ = 6.

## III Results

**Fig. 2:**
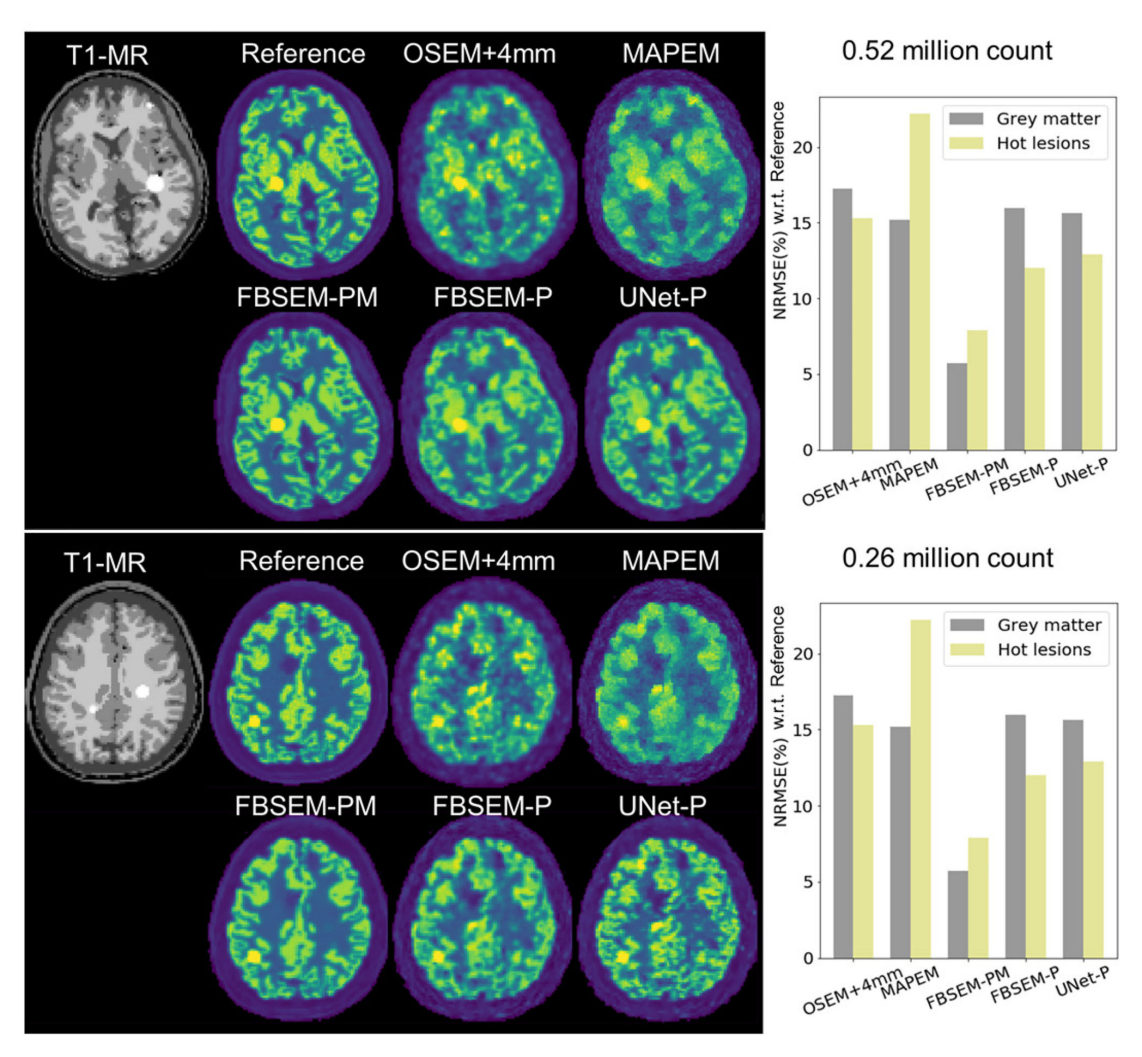
Evaluation of the FBSEM and U-Net networks, trained with a 1M-count dataset, on test datasets with half and quarter dose compared with smoothed OSEM and MR-guided MAPEM reconstructions. NRMSE performance of the methods are shown for hot lesions and grey matter.

**Fig. 3:**
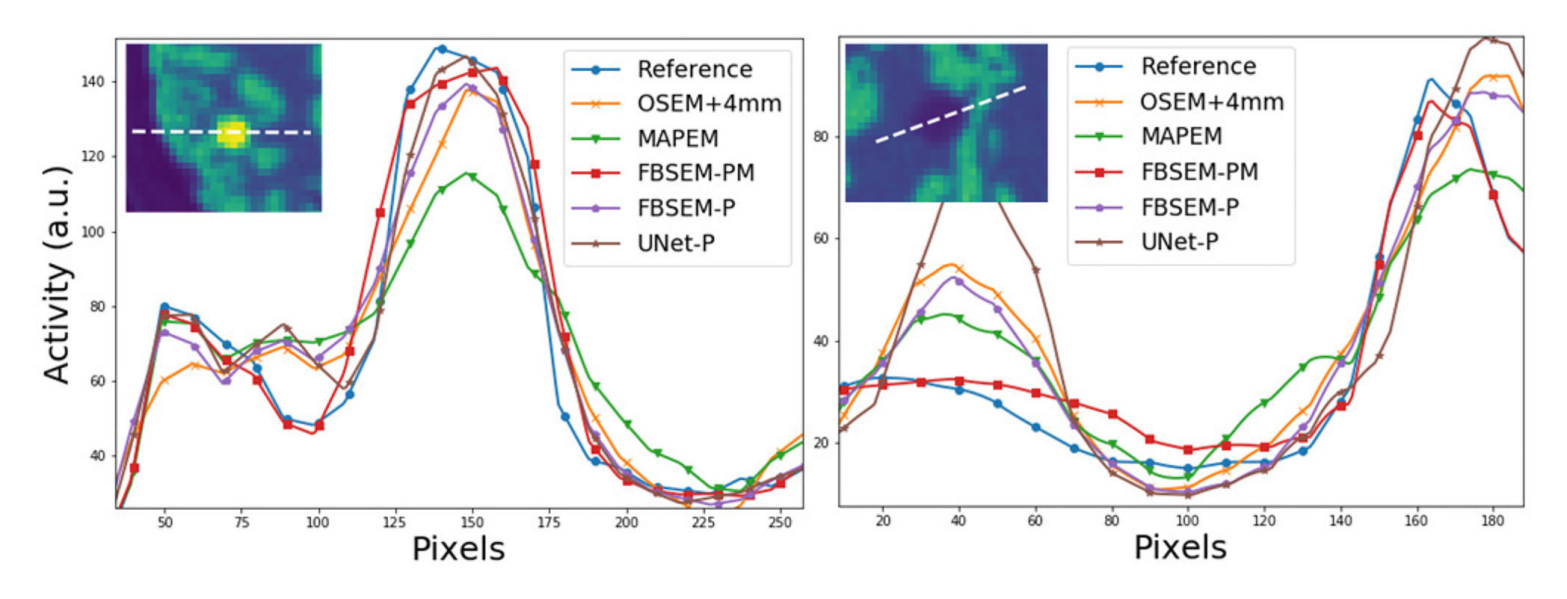
Comparison of activity profiles through cold and hot lesions of the 0.26M-count dataset.

Fig. 2 shows the reconstruction results from the proposed network trained on PET-MR (FBSEM-PM) and PET-only (FBSEM-P) data in comparison with U-Net denoiser trained on PET-only OSEM images (UNet-P), 4-mm-smoothed OSEM and MR-guided MAPEM for half-dose and quarter-dose test data samples. NRMSE performance of the methods is also shown for grey matter and hot lesions. For both datasets, OSEM images are affected by noise and partial volume effects; MAPEM reduces noise at the cost of suppressing lesions (highest NRMSE). FBSEM-PM accuartely reconstructs the underlying activity given that there are multiple mismatched PET-MR lesions (lowest NRMSE). For both datasets, the FBSEM-P and UNet-P have roughly comparable NRMSEs, however, for the 0.26M-count dataset, FBSEM-P shows lower noise in the low-uptake white matter regions. Fig. 3 compares activity profiles through the hot and cold lesions of the 0.26M-count dataset. As shown, FBSEM-PM follows reference profiles faithfully for both hot lesion and low-contrast cold one; and FBSEM-P and UNet-P perform better or similar to OSEM method.

**Fig. 4:**
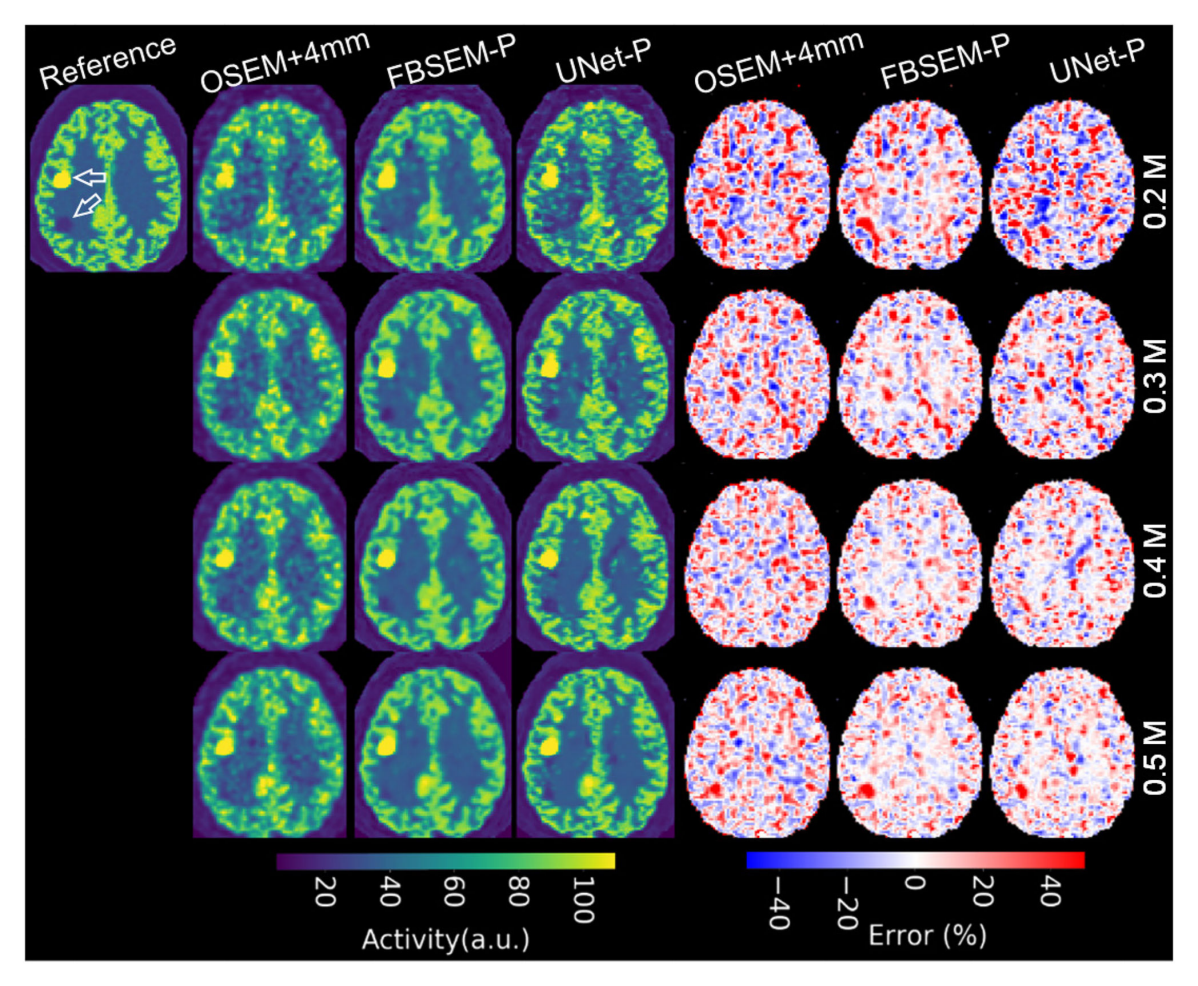
Performance of FBSEM-P and UNet-P, for different dose levels of a test dataset with hot and cold lesions. Error maps show voxel-wise relative differences to the reference image.

To demonstrate the added value of MoDL reconstruction compared with post-reconstruction DL denoising, the FBSEM-P and UNet-P were evaluated for a test dataset with decreasing level of dose. Fig. 4 shows the reconstruction results for count levels from 0.2 to 0.5 M counts. OSEM images and error maps have also been shown. Note both networks were trained on the same dataset and the regularization parameter of FBSEM-P was not modified even though it had been trained on a 1M-count dataset. As the count level is decreased, the results show the outperformance of FBSEM-P for reducing noise and noise-induced cold spots visible in both OSEM and UNet-P images. Note that the simulations were performed with different noise realizations, hence noise pattern and the cold lesion’s detectability are different between the studied count levels.

**Fig. 5:**
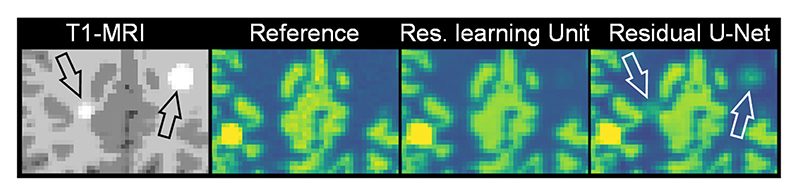
Performance of FBSEM-PM net, with residual learning unit (shown in Fig.1) and a residual U-Net, over mismatched PET and MR lesions.

The performance of FBSEM was also evaluated using a residual U-Net as a deep prior. For FBSEM-P net, it achieved a comparable performance to a residual learning unit, however, as shown in Fig. 5, for FBSEM-PM, this prior introduced MR-unique lesions into PET images, while preserving PET lesions. We also compared UNet-P post-reconstruction denoiser with a learning-unit denoiser, the results (not shown here) showed that a U-Net gives a better performance.

## IV Discussion and Conclusion

Compared to the recently proposed BCD net [8], our proposed net has a number of advantages: i) similar to RNNs, model parameters are shared across all reconstruction states, hence not only the number of trainable parameters is notably lower but also the network keeps track of dependencies between image estimates and of how noise is progressively intensified. This might explain its outperformance over the post-reconstruction U-Net denoiser. ii) The regularization parameter is trained; and our results showed that the trained network performs acceptably even for datasets with different noise levels. iii) The input of FBSEM net is initialized with a uniform image, and all model parameters are randomly initialised. In fact, for *N*_*sub*_ = 1, the *trained* FBSEM net can be monotonically convergent, depending on the convexity of the learned prior. In future work, the network will be upgraded using a convergent OS technique such as RAMLA. In addition, owing to a lower number of parameters and the fact that backpropagation passes only through the regularization and fusion modules, a relatively plateaued training loss was obtained after only 50 epochs. This will be further investigated in future work, together with 3D simulations and *in-vivo* evaluations.
